# Multi-Tissue Metabolomics Reveals Metabolic Signatures Associated with Lipid Partitioning Between Abdominal Fat and Egg Yolk in Laying Hens

**DOI:** 10.3390/ani16121812

**Published:** 2026-06-11

**Authors:** Wenxin Zhang, Ronglang Cai, Fangren Lan, Guiqin Wu, Guangqi Li, Yiyuan Yan, Ning Yang, Congjiao Sun

**Affiliations:** 1 State Key Laboratory of Animal Biotech Breeding, Frontier Science Center of Molecular Design Breeding, China Agricultural University, Beijing 100193, China; 2 National Engineering Laboratory for Animal Breeding, Key Laboratory of Animal Genetics, Breeding and Reproduction, Ministry of Agriculture and Rural Affairs, China Agricultural University, Beijing 100193, China; 3 Department of Animal Genetics and Breeding, College of Animal Science and Technology, China Agricultural University, Beijing 100193, China; 4 Beijing Engineering Research Centre of Layer, Beijing 101206, China; 5 Beijing Huadu Yukou Poultry Industry Co., Ltd., Beijing 101206, China

**Keywords:** laying hens, abdominal fat deposition, egg yolk lipid deposition, multi-tissue metabolomics, bidirectional GSMR, lipid partitioning

## Abstract

Fat deposition in laying hens involves lipid synthesis, transport, storage, and allocation across multiple tissues, yet the metabolic links between abdominal fat (AF) and egg yolk (EY) lipid deposition remain unclear. Here, we integrated whole-genome resequencing data with untargeted metabolomic profiles from the liver, duodenum, ileum, cecum, and serum of 248 purebred Rhode Island Red hens at 100 weeks of age. We estimated metabolite-explained variance (*me*^2^) for 22 fat deposition-related traits, evaluated metabolite heritability, and combined Spearman correlation analysis with bidirectional generalized summary-data-based Mendelian randomization (GSMR) to identify shared metabolites and pathways associated with AF and EY traits. The *me*^2^ showed clear tissue specificity, with the liver, serum, and duodenum showing significant explanatory signals for 77.3% (17/22), 72.7% (16/22), and 68.2% (15/22) of fat-related traits. Liver-, AF-, and body weight-related traits showed stronger metabolomic explanatory signals, with significant proportions of 71.4–100.0%, 28.6–100.0%, and 100.0% across tissues, respectively, whereas EY-related traits showed more restricted and tissue-specific associations (0–33.3%). Correlation analysis identified liver-enriched AF–EY shared metabolites (e.g., NADPH, cholesteryl sulfate, N6,N6,N6-trimethyllysine), most of which showed opposite association patterns between AF- and EY-related traits. Bidirectional GSMR prioritized 20 candidate metabolites with opposite putative effects on AF and EY traits, including CDP-choline, phosphorylcholine, and allantoin. Pathway integration highlighted fructose/mannose metabolism, glycerophospholipid metabolism, ABC transporters, folate/one-carbon metabolism, amino acid metabolism, and energy metabolism as core components of the AF–EY shared network. These findings reveal tissue-specific and shared metabolic bases of fat deposition and identify candidate metabolic signatures associated with lipid partitioning between abdominal fat and egg yolk in laying hens.

## Simple Summary

Excessive abdominal fat deposition reduces production efficiency in laying hens, whereas egg yolk lipid deposition is essential for egg quality and embryo development. Because both traits depend on lipid synthesis, transport, and allocation, understanding their shared metabolic basis may help improve lipid utilization in poultry. In this study, we integrated whole-genome resequencing data with metabolomic profiles from the liver, duodenum, ileum, cecum, and serum of 248 Rhode Island Red laying hens at 100 weeks of age. We evaluated the genetic contribution of metabolites, quantified the proportion of phenotypic variation explained by tissue-specific metabolomic profiles, and identified candidate metabolites associated with abdominal fat and egg yolk lipid deposition. The liver metabolome showed the strongest associations with lipid deposition-related traits. Several metabolites, including sugar phosphate metabolites, NADPH, N6,N6,N6-trimethyllysine, cytidine 5′-diphosphocholine (CDP-choline), phosphorylcholine, and allantoin, were identified as candidate metabolic signatures associated with lipid allocation between abdominal fat and egg yolk. Pathway analyses highlighted carbohydrate metabolism, glycerophospholipid metabolism, one-carbon metabolism, amino acid metabolism, and energy metabolism. These findings improve our understanding of lipid allocation in laying hens and provide candidate metabolic indicators for future nutritional, genetic, and functional studies.

## 1. Introduction

Fat deposition is a key biological and economic trait in laying hens, influencing production efficiency, animal health, carcass value, and egg quality. Excessive abdominal fat deposition not only reduces feed conversion efficiency and carcass grade [1,2,3], but also increases environmental burden [4]. Conversely, the lipid content and composition of egg yolk directly determine egg nutritional value and consumer preference. Therefore, elucidating the metabolic regulatory mechanisms underlying fat deposition is of both theoretical and practical significance, aiming to reduce fat accumulation, improve production efficiency, and achieve coordinated enhancement of product quality.

Fat deposition is inherently a multi-tissue coordinated process involving lipid synthesis, transport, storage, and allocation [5,6], yet most previous studies have investigated lipid metabolism-related metabolite changes only in single tissues such as liver, adipose tissue, or blood. In poultry, the liver is the primary site of de novo fatty acid synthesis [7,8]. Hepatically synthesized triglycerides are secreted into circulation as lipoproteins, with one fraction transported to abdominal fat for storage and another deposited into egg yolk [5,9,10,11]. Here, lipid partitioning refers to the differential allocation of lipids synthesized or transported through hepatic and circulating metabolic pathways among competing physiological destinations, particularly storage in abdominal fat and deposition into egg yolk. Thus, hepatic lipid metabolism is mechanistically linked to liver lipid deposition, fat accumulation, and egg yolk lipid deposition [12]. Extending the laying cycle to 90–100 weeks of age improves resource-use efficiency and environmental sustainability, but this extension depends on maintaining egg quality, hen health, welfare, and nutritional support [13]. However, as the laying period extends, hens become increasingly susceptible to metabolic imbalance, characterized by excessive abdominal fat deposition and hepatic lipid accumulation, which may ultimately impair laying persistency, liver health, and egg quality [14,15,16,17]. Recent lipidomic evidence further indicates that fatty liver lesions can alter the egg yolk lipid profile and thus affect the nutritional value of eggs [18]. Notably, despite sharing a common dependence on hepatic lipid synthesis, lipoprotein assembly, circulating lipid transport, and tissue-specific lipid uptake, these traits—abdominal fat deposition, hepatic lipid accumulation, and egg yolk lipid deposition—have typically been studied separately.

Metabolomics provides a direct readout of metabolic status and is therefore well suited for linking complex phenotypes to biological processes [19,20]. Recent metabolomic and lipidomic studies in laying hens have shown that egg yolk metabolites are influenced by breed, diet, and lipid metabolic status, with pathways such as glycerolipid and glycerophospholipid metabolism associated with egg yolk lipid deposition [21,22]. In chickens, serum metabolome-wide association studies have further demonstrated that metabolite abundance has a detectable genetic basis [23], highlighting the value of integrating metabolomics with genomic information to identify genetically regulated metabolic features. Nevertheless, most previous studies have focused on single tissues, blood metabolites, or specific nutritional interventions [22,23,24], and consequently, the extent to which multi-tissue metabolites explain phenotypic variation in fat deposition-related traits during the extended laying period remains largely unclear. In addition, the metabolic links between abdominal fat deposition and egg yolk lipid deposition are poorly understood. From a breeding perspective, reducing excessive abdominal fat while maintaining favorable egg yolk lipid deposition is an important but challenging goal, requiring a deeper understanding of whether these two types of traits share common metabolites, metabolic pathways, or genetically influenced metabolic mediators. Although correlation-based metabolomic analyses can identify phenotype-associated metabolites, they cannot distinguish whether these metabolites act as potential causal mediators, downstream consequences, or merely correlated metabolic markers. Generalized summary-data-based Mendelian randomization (GSMR) [25], which uses GWAS summary statistics to evaluate genetically inferred directional associations between traits, provides an effective framework for prioritizing candidate metabolites associated with phenotypes of interest. However, few studies have integrated multi-tissue metabolomics, metabolite heritability estimation, and bidirectional GSMR analysis to investigate the relationship between abdominal fat deposition and egg yolk lipid deposition in laying hens.

To address these gaps, this study integrated whole-genome resequencing data with metabolomic profiles from liver, duodenum, ileum, cecum, and serum of hens during the extended laying period. We systematically evaluated the proportion of phenotypic variance in 22 fat deposition-related traits, including hepatic lipid traits, abdominal fat-related traits, egg yolk-related traits, and body weight-related traits, explained by tissue-specific metabolomic profiles, as well as the heritability of metabolite abundance. We further performed bidirectional GSMR analysis to prioritize metabolites associated with abdominal fat and egg yolk lipid deposition. By integrating these results with correlation analysis and KEGG enrichment network analysis, we focused on identifying metabolites shared between abdominal fat and egg yolk traits that showed opposite effect directions, as well as their core pathways. This study aims to uncover candidate metabolic signatures associated with lipid allocation between abdominal fat and egg yolk, thereby providing potential biomarkers and theoretical insights for future studies on body fat deposition and egg quality in chickens.

## 2. Materials and Methods

### 2.1. Ethics Statement

All animal experiments were conducted in accordance with protocols approved by the Institutional Animal Care and Use Committee of China Agricultural University, China (Approval No. AW02205202-01, approved on 20 February 2025).

### 2.2. Experimental Population, Phenotype Records, and Sample Collection

The study population comprised 248 purebred Rhode Island Red laying hens from Beijing Huadu Yukou Poultry Industry Co., Ltd. (Pinggu, Beijing, China). Details of animal management, phenotypic recording, and the collection of major tissues have been reported previously [26]. Briefly, hens were individually housed under standardized conditions and provided ad libitum access to a corn–soybean meal-based diet and water. Fat deposition-related phenotypes used in this study included liver-, abdominal fat-, egg yolk-, and body weight-related traits measured around 100 weeks of age.

At 100 weeks of age, whole blood was collected from the wing vein for whole-genome resequencing, and eggs produced during the measurement period were collected for biochemical assessment of egg yolk traits. Hens were then euthanized by cervical dislocation and dissected. Liver tissue and abdominal fat were collected, and liver weight (LW), abdominal fat weight (AFW), and abdominal fat percentage (AFP) were determined as described previously [26].

For metabolomic analysis, liver, duodenal contents, ileal contents, cecal contents, and serum samples were used. Tissue and intestinal content sampling followed the same collection and storage procedures described in our previous work [26], with samples immediately frozen in liquid nitrogen and stored at −80 °C. Serum samples were additionally prepared by collecting approximately 5 mL of whole blood into tubes without anticoagulant, followed by natural serum separation and centrifugation at 3000× *g* for 15 min. The serum supernatant was collected and stored at −20 °C until metabolomic profiling.

### 2.3. Biochemical Measurements and Phenotypic Definitions

Biochemical phenotypes for liver, abdominal fat, and egg yolk samples were obtained from the same experimental population and measured using the protocols described previously [27]. These phenotypes included dry matter, triglyceride, free fatty acid, and total cholesterol-related indicators: liver dry matter content (LDM), liver triglyceride content (LTG), liver free fatty acid content (LFFA), liver total cholesterol content (LTC), abdominal fat dry matter content (AFDM), abdominal fat triglyceride content (AFTG), abdominal fat free fatty acid content (AFFFA), egg yolk dry matter content (EYDM), egg yolk triglyceride content (EYTG), and egg yolk free fatty acid content (EYFFA). Briefly, dry matter content was determined using an oven-drying method for solid samples according to the Chinese National Standard GB 5009.3-2016 (https://www.svscr.cz/wp-content/files/obchodovani/GB_5009.3-2016_Moisture_in_Foods.pdf, accessed on 9 June 2026), whereas triglyceride, free fatty acid, and total cholesterol levels were quantified using commercial colorimetric assay kits following the manufacturers’ instructions. All samples were assayed in duplicate.

To distinguish directly measured percentage traits from derived mass traits, dry matter percentage traits in abdominal fat, egg yolk, and liver were denoted as AFDMP, EYDMP, and LDMP, respectively. Dry matter mass was calculated as tissue weight multiplied by the corresponding dry matter proportion, and moisture mass was calculated as tissue weight minus dry matter mass. Accordingly, AFDM, EYDM, and LDM denote dry matter mass, whereas AFMC, EYMC, and LMC denote moisture mass in the corresponding tissues. AFW, EYW, and LW represent abdominal fat weight, egg yolk weight, and liver weight, respectively. Body weight-related phenotypes included mBW100, defined as the average body weight measured at the beginning and end of the 99–100-week experimental period, and BW100, defined as the body weight recorded at the end of this period.

### 2.4. Untargeted LC–MS/MS Metabolomic Profiling and Data Processing

For metabolomic analysis, liver tissue as well as duodenal, ileal, and cecal content samples (25 ± 1 mg) were mixed with ceramic beads and 500 μL extraction solvent (methanol:acetonitrile:water = 2:2:1, *v*/*v*) containing isotopically labeled internal standards. After vortexing for 30 s, the samples were homogenized at 35 Hz for 4 min and sonicated for 5 min in an ice-water bath at 4 °C. The homogenization–sonication cycle was repeated thrice. Samples were then incubated at −40 °C for 1 h to precipitate proteins and centrifuged at 12,000 rpm (RCF = 13,800× *g*, R = 8.6 cm) for 15 min, at 4 °C. The supernatants were carefully transferred to fresh glass autosampler vials for subsequent LC–MS/MS analysis. Quality control (QC) samples were prepared by pooling equal aliquots of the supernatants from all the individual samples.

Polar metabolites were analyzed using an ultra-high-performance liquid chromatography system (Vanquish, Thermo Fisher Scientific, Waltham, MA, USA) coupled to an Orbitrap Exploris 120 mass spectrometer (Thermo Fisher Scientific, Waltham, MA, USA) and equipped with a Waters ACQUITY UPLC BEH Amide column (2.1 mm × 50 mm, 1.7 μm; Waters Corporation, Milford, MA, USA). The mobile phases consisted of 25 mmol/L ammonium acetate and 25 mmol/L ammonium hydroxide in water (pH 9.75) as solvent A, and acetonitrile as solvent B. The autosampler was maintained at 4 °C, and the injection volume was 2 μL. MS and MS/MS data were acquired in information-dependent acquisition (IDA) mode using Xcalibur software, https://www.thermofisher.com/ro/en/home/industrial/mass-spectrometry/liquid-chromatography-mass-spectrometry-lc-ms/lc-ms-software/lc-ms-data-acquisition-software/xcalibur-data-acquisition-interpretation-software.html, accessed on 9 June 2026 (Thermo Fisher Scientific, Waltham, MA, USA); the acquisition software continuously evaluated full-scan MS spectra to trigger MS/MS acquisition. The electrospray ionization (ESI) source parameters were set as follows: sheath gas, 50 Arb; auxiliary gas, 15 Arb; capillary temperature, 320 °C; full-scan resolution, 60,000; MS/MS resolution, 15,000; and stepped normalized collision energies of 20, 30, and 40 eV. The spray voltage was set to +3.8 kV in positive ion mode and −3.4 kV in negative ion mode.

Raw data files were first converted to mzXML format using ProteoWizard. Peak detection, extraction, alignment, and integration were then performed using in-house R scripts based on the XCMS package. After peak detection, alignment, and integration, relative metabolite abundance was calculated based on integrated peak signal intensity normalized to the corresponding internal standards. Metabolite annotation was performed using in-house R workflows together with BiotreeDB (v3.0) [28]. For each tissue, metabolite features with non-missing MS2 scores were first retained as MS/MS-supported metabolite features. These features were then further filtered by detection rate, and only features detected in more than 20% of samples within each tissue were retained for downstream analyses. No additional samples were excluded unless they showed obvious technical failure during sample preparation or LC–MS/MS acquisition. After filtering, the metabolite abundance matrix was transposed into an individual-by-metabolite format, and feature intensities were standardized using Z-score transformation with the scale function in R (v4.4.1).

### 2.5. Heritability of Metabolites and Metabolite-Explained Variance Estimation

For each tissue, metabolites with a detection rate greater than 20% were retained. Metabolite abundance matrices were transposed to an individual-by-metabolite format, followed by Z-score standardization. The Shapiro–Wilk test was used to assess normality for each metabolite, and metabolites that deviated from normality were transformed using rank-based inverse normal transformation, with a small random perturbation added when necessary to reduce tied ranks. The normalized metabolite matrices were then used for heritability and metabolite-explained variance analyses. SNP-based heritability was estimated separately for metabolites detected in the liver, duodenum, ileum, cecum, and serum using restricted maximum likelihood (REML) implemented in GCTA (v1.93.2) [29]. REML was chosen because it provides an appropriate variance-component framework for estimating SNP-based heritability in a linear mixed model. In this framework, the genomic relationship matrix (GRM) is fitted as a random effect, allowing the variance in metabolite abundance to be partitioned into the component captured by genome-wide SNPs included in the GRM and the residual component, while accounting for genetic relatedness among individuals. The GRM was constructed from 6,524,337 quality-controlled SNPs, and the top 10 genetic principal components derived from independent SNPs were included as fixed-effect covariates to account for population structure. For each metabolite, the heritability estimate, standard error, and corresponding *p* value were obtained from the REML analysis. We also estimated the proportion of phenotypic variance explained by tissue-specific metabolomic profiles, defined as metabolite-explained variance (${me}^{2}$). For each tissue, a metabolite relationship matrix was constructed from normalized metabolite abundance data and fitted as a random effect in a mixed model:

*y* = X*β* + *m* + *ε* (1)

where *y* is the vector of phenotypic values for a given fat deposition-related trait, X is the design matrix for fixed-effect covariates including the top 10 genetic principal components, and *β* is the corresponding vector of fixed-effect estimates. The term m represents the random effect attributable to the tissue-specific metabolomic profile, assumed to follow *N* (0, $M\sigma_{m}^{2}$), where M is the metabolite relationship matrix and $\sigma_{m}^{2}$ is the metabolite-associated variance component. The term *ε* is a vector of residual errors. The element of the metabolite relationship matrix between individuals (i) and (j) was calculated as:

(2) $$M_{ij}=\frac{1}{N_{s}}\sum_{k=1}^{N_{s}}\frac{{(x}_{ik}-\bar{x_{k}}){(x}_{jk}-\bar{x_{k}})}{\sigma_{k}^{2}}$$

where *M*_ij_ represents the estimated metabolomic similarity between individuals i and j; *x_ik_* and *x_jk_* denote the normalized abundances of metabolite *k* in individuals *i* and *j*, respectively; $\bar{x_{k}}$ is the mean abundance of metabolite *k* across individuals; $\sigma_{k}^{2}$ is the variance of metabolite *k*; and *N_s_* is the total number of metabolites used to construct the tissue-specific metabolite relationship matrix. The metabolite-explained variance was calculated as:

(3) $${me}^{2}=\frac{\sigma_{m}^{2}}{\sigma_{m}^{2}+\sigma_{e}^{2}}$$

where ${me}^{2}$ represents the proportion of total phenotypic variance explained by the metabolite abundance profile of a given tissue. This analysis was performed separately for the liver, duodenum, ileum, cecum, and serum to assess tissue-specific contributions of metabolomic profiles to fat deposition-related phenotypes.

### 2.6. Genome-Wide Association Analysis

Genotype data used for GWAS were obtained from whole-genome resequencing and processed as described previously [26], yielding 6,524,337 quality-controlled SNPs from 248 individuals across 39 chromosomes. GWAS for fat deposition-related phenotypes were performed using a linear mixed model in GEMMA (v0.98.4) [30]. SNP effects were tested while accounting for relatedness among individuals through a random polygenic effect. The model included an intercept and the top five genetic principal components as fixed effects to control for population stratification, and SNP significance was assessed using likelihood ratio tests. The effective number of independent markers was estimated using the R package simpleM [31] (https://www.r-project.org/) to account for linkage disequilibrium among genome-wide SNPs. This yielded 226,205 effective independent tests, corresponding to a genome-wide significance threshold of 2.21 × 10^−7^ (0.05/226,205) and a suggestive threshold of 4.42 × 10^−6^ (1/226,205).

Metabolite GWAS was conducted for metabolites detected in more than 20% of samples from the liver, duodenum, ileum, cecum, and serum. The normalized metabolite abundance values described above were used as metabolite phenotypes for association testing. Association testing was performed using fastGWA [32] in GCTA, with metabolite abundance treated as the dependent variable and SNP genotypes as predictors. A sparse GRM derived from the full-density GRM using a relatedness cutoff of 0.05 was used to account for genetic relatedness. The same significance thresholds were applied to all metabolite GWAS analyses.

### 2.7. Bidirectional GSMR Analysis Between Metabolites and Phenotypes

To evaluate genetically inferred directional associations between metabolite abundance and fat deposition-related phenotypes, we performed bidirectional generalized summary-data-based Mendelian randomization (GSMR) [25] analyses using metabolite GWAS and phenotype GWAS summary statistics. Metabolites from five tissues or sample types, including liver, duodenum, ileum, cecum, and serum, were analyzed separately. For each metabolite and phenotype, genome-wide association summary statistics were first generated as described above. Instrumental variants were selected from the corresponding exposure GWAS using the suggestive association threshold of *p* < 4.42 × 10^−6^. To ensure relative independence among instruments, selected SNPs were pruned for linkage disequilibrium using an *r*^2^ < 0.2 threshold. HEIDI-outlier filtering was then applied to reduce the influence of variants with evidence of horizontal pleiotropy. GSMR analyses were conducted in both directions. In the metabolite-to-phenotype direction, metabolite abundance was treated as the exposure and fat deposition-related phenotypes as outcomes. In the phenotype-to-metabolite direction, phenotypes were treated as exposures and metabolite abundance as outcomes. For each metabolite–phenotype pair, GSMR effect estimates, standard errors, and *p* values were obtained for both directions. Associations reaching nominal significance in either direction were retained for downstream integrative analyses.

### 2.8. Statistical Analysis

The normality of each phenotype was assessed using the Shapiro–Wilk test in R (v4.4.1). Phenotypes that deviated from normality were transformed using rank-based inverse normal transformation and then re-tested for normality. All correlation analyses were performed in R using the cor.test function. KEGG pathway enrichment analysis was performed using the enricher function in the clusterProfiler package [33]. The resulting *p* values were adjusted for multiple tests using the Benjamini–Hochberg method, and significantly enriched metabolic pathways were identified based on the adjusted significance levels. Pathway similarity was quantified using the Jaccard index based on shared mapped metabolites, and an enrichment network was constructed to visualize relationships among enriched pathways.

## 3. Results

### 3.1. Multi-Tissue Metabolite-Explained Variance Reveals Tissue-Specific Metabolic Bases for Fat Deposition

**Figure 1:**
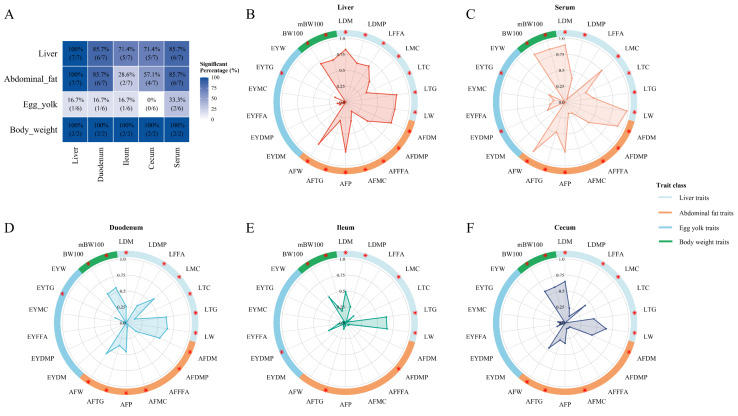
Tissue-specific metabolite-explained variance (*me*^2^) of fat deposition-related phenotypes. (**A**) Proportion of phenotypes with significant metabolite-explained variance across five tissues and four trait classes. Numbers indicate the percentage of significant phenotypes, with counts shown in parentheses. (**B**–**F**) Radar plots showing phenotype-specific *me*^2^ estimates in the liver (**B**), serum (**C**), duodenum (**D**), ileum (**E**), and cecum (**F**). Each spoke represents one phenotype, and the distance from the center indicates the magnitude of *me*^2^. Red asterisks denote statistically significant *me*^2^ estimates (*p* < 0.05), and the outer colored ring indicates trait class. Liver-related traits (L): LW, liver weight; LTC, liver total cholesterol content; LTG, liver triglyceride content; LFFA, liver free fatty acid content; LDMP, liver dry matter percentage; LDM, liver dry matter mass; LMC, liver moisture mass. Abdominal fat-related traits (AF): AFW, abdominal fat weight; AFP, abdominal fat percentage; AFTG, abdominal fat triglyceride content; AFFFA, abdominal fat free fatty acid content; AFDMP, abdominal fat dry matter percentage; AFDM, abdominal fat dry matter mass; AFMC, abdominal fat moisture mass. Egg yolk-related traits (EY): EYW, egg yolk weight; EYTG, egg yolk triglyceride content; EYFFA, egg yolk free fatty acid content; EYDMP, egg yolk dry matter percentage; EYDM, egg yolk dry matter mass; EYMC, egg yolk moisture mass. Body weight-related traits (BW): BW100, body weight at 100 weeks of age; mBW100, mean body weight during weeks 99–100.

Descriptive statistics for fat-related phenotypes are provided in Supplementary Table S1 and have been described in detail in our previous study [27]. The 22 phenotypes were grouped into four categories: liver-related traits (L), including LW, LFFA, LTG, LTC, LDMP, LDM, and LMC; abdominal fat-related traits (AF), including AFW, AFP, AFFFA, AFTG, AFDMP, AFDM, and AFMC; egg yolk-related traits (EY), including EYW, EYFFA, EYTG, EYDMP, EYDM, and EYMC; and body weight-related traits (BW), including BW100 and mBW100. The metabolomic dataset included 248 liver, 248 duodenum, 235 ileum, 248 cecum, and 131 serum samples. After filtering with a detection rate greater than 20%, 1826, 2072, 2368, 2487, and 1995 metabolites were retained in the liver, duodenum, ileum, cecum, and serum, respectively, of which 400 (21.91%), 374 (18.05%), 437 (18.45%), 459 (18.46%), and 397 (19.90%) were annotated with KEGG IDs. Therefore, KEGG pathway enrichment analyses were based on the annotated metabolite subset and should be interpreted as pathway-level signals from currently annotated metabolites rather than the complete metabolome. To assess the contribution of tissue-specific metabolic profiles to fat deposition-related phenotypic variation, we introduced a new concept of metabolite-explained variance (*me*^2^) to estimate the proportion of variance explained by metabolite matrices from each tissue. Higher *me*^2^ values indicate a greater contribution of the corresponding tissue metabolome to phenotypic variation. Then *me*^2^ was estimated separately in the liver, duodenum, ileum, cecum, and serum for each fat deposition-related trait (Figure 1B–F; Supplementary Table S2).

The explanatory capacity of metabolomic profiles differed markedly among tissues and trait classes. At the tissue level, the liver showed the highest proportion of traits with significant *me*^2^ estimates, reaching 77.3% (17/22), followed by serum at 72.7% (16/22) and duodenum at 68.2% (15/22). In contrast, lower proportions were observed in the cecum and ileum, with 50.0% (11/22) and 45.5% (10/22) of traits showing significant *me*^2^, respectively (Figure 1A). At trait category level, liver (L)- and abdominal fat (AF)-related traits showed relatively broad metabolomic explanatory signals, with significant *me*^2^ proportions of 71.4–100.0% and 28.6–100.0% across tissues, respectively. Body weight (BW)-related traits showed significant *me*^2^ in all tissues, whereas egg yolk (EY)-related traits showed more restricted and tissue-specific signals, with significant *me*^2^ proportions ranging from 0 to 33.3% (Figure 1A). Consistently, the liver metabolomic profile explained a high proportion of variation in several liver lipid and abdominal fat traits, including LDM, LTG, LW, AFW, AFP, and AFDM, with *me*^2^ estimates of approximately 0.78–0.83 (Figure 1B; Supplementary Table S2). Serum also showed strong explanatory capacity for LW, LDM, AFW, and AFDM, with *me*^2^ estimates of approximately 0.90–0.98 (Figure 1C; Supplementary Table S2). Duodenal metabolites showed moderate explanatory signals for several liver/abdominal fat traits, but ileal/cecal signals were more trait-restricted. (Figure 1D–F; Supplementary Table S2). These results indicate that liver and serum metabolomic profiles capture major metabolic variation related to hepatic lipid status and abdominal fat deposition.

### 3.2. Heritability of Multi-Tissue Metabolites

**Figure 2:**
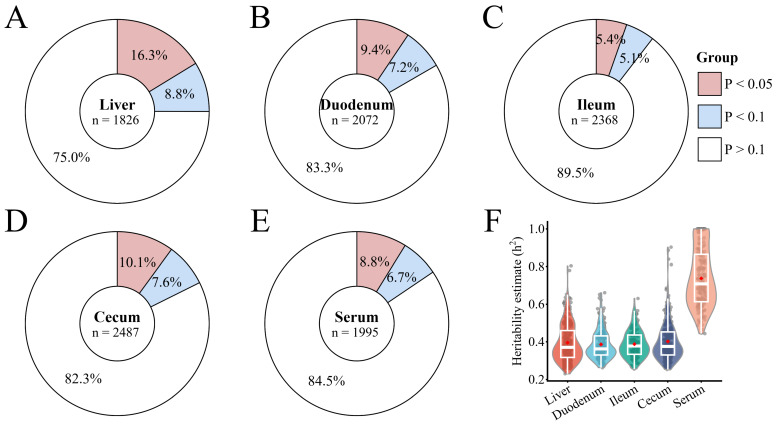
Tissue-specific heritability patterns of multi-tissue metabolites. (**A**–**E**) Donut charts showing the distribution of heritability (*h*^2^) estimates for metabolites detected in the liver (**A**), duodenum (**B**), ileum (**C**), cecum (**D**), and serum (**E**). Metabolites with a detection rate greater than 20% were included in the analysis. Metabolites were grouped according to the significance level of heritability estimates: significant heritability (*p* < 0.05), suggestive heritability (0.05 ≤ *p* < 0.10), and non-significant heritability (*p* ≥ 0.10). Percentages indicate the proportion of metabolites in each significance category within each tissue. The total number of metabolites analyzed in each tissue is shown in the center of each donut chart. (**F**) Violin plots overlaid with boxplots showing the distribution of *h*^2^ estimates for metabolites with significant heritability (*p* < 0.05) in the liver, duodenum, ileum, cecum, and serum. Each point represents one metabolite. The box indicates the interquartile range, the horizontal line indicates the median, and the red diamond indicates the mean *h*^2^ value for each tissue.

To further evaluate the genetic basis of multi-tissue metabolite levels, we estimated the heritability of all detected metabolites with a detection rate greater than 20% in the liver, duodenum, ileum, cecum, and serum. Using the whole-genome resequencing data generated in our previous study [26], 6,524,337 quality-controlled SNPs from 248 individuals across 39 chromosomes were used to construct the genomic relationship matrix for metabolite heritability estimation. Metabolites were grouped according to the significance level of heritability estimates: *p* < 0.05, 0.05 ≤ *p* < 0.10, and *p* ≥ 0.10. Overall, the proportion of metabolites with significant heritability varied across tissues (Figure 2A–E; Supplementary Table S3). The liver showed the highest proportion of significantly heritable metabolites, with 297 of 1826 metabolites reaching *p* < 0.05 (16.3%), followed by the cecum (250/2487, 10.1%), duodenum (195/2072, 9.4%), serum (176/1995, 8.8%), and ileum (127/2368, 5.4%) (Figure 2A–E; Supplementary Table S3). Thus, the liver contained the largest fraction of metabolites influenced by genetic variation. However, among significantly heritable metabolites, serum showed the highest average *h*^2^ estimate (mean *h*^2^ = 0.737; median *h*^2^ = 0.708), whereas the duodenum showed the lowest mean *h*^2^ (0.386) (Figure 2F; Supplementary Table S3). The corresponding mean *h*^2^ estimates in the cecum, liver, and ileum were 0.402, 0.395, and 0.388, respectively (Figure 2F; Supplementary Table S3). When suggestive heritability signals were also considered (*p* < 0.10), approximately 25.1% of liver metabolites showed evidence of genetic contribution, which was higher than the corresponding proportions in the duodenum (16.6%), ileum (10.5%), cecum (17.7%), and serum (15.5%) (Figure 2A–E; Supplementary Table S3). These results suggest that the levels of some metabolites are influenced by genetic factors and that this genetic contribution varies substantially among tissues, with liver metabolites showing the broadest genetic regulatory features and serum metabolites showing the strongest average heritability among significant metabolites.

### 3.3. Key Metabolites and Pathways Shared Between Abdominal Fat Deposition and Egg Yolk Lipid Deposition

**Figure 3:**
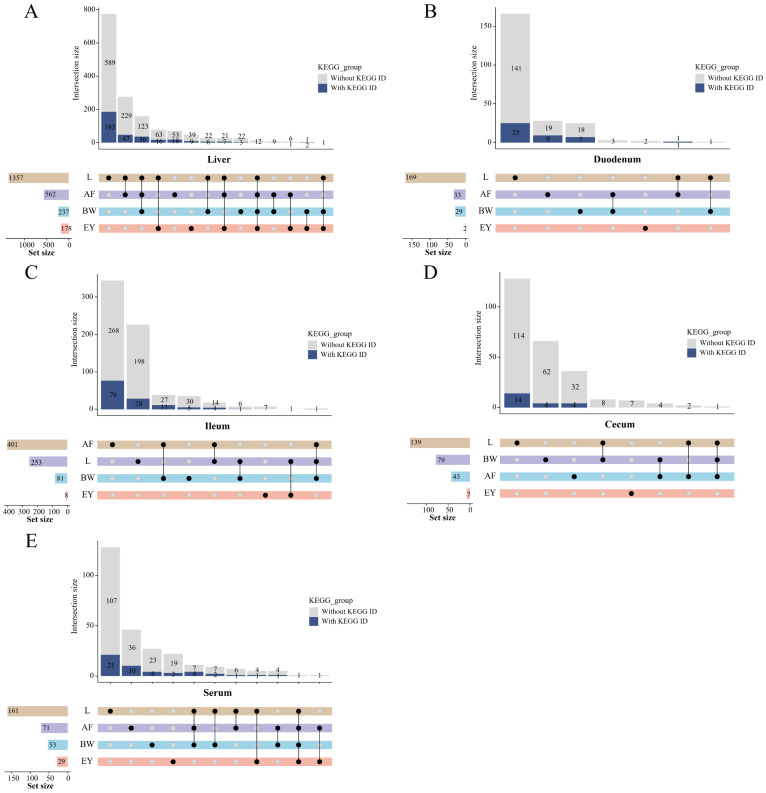
Tissue-specific intersections of metabolites significantly correlated with fat deposition-related trait classes. (**A**–**E**) UpSet plots showing metabolites significantly correlated with liver-related traits (L), abdominal fat-related traits (AF), egg yolk-related traits (EY), and body weight-related traits (BW) in the liver (**A**), duodenum (**B**), ileum (**C**), cecum (**D**), and serum (**E**). Significant correlations were identified by Spearman rank correlation analysis after FDR correction (*P*_adj_ < 0.05). Left horizontal bars indicate the total number of significant metabolites associated with each trait class. Black connected dots indicate intersections among trait classes. Vertical bars indicate the number of metabolites within each intersection. Dark blue segments represent metabolites annotated with KEGG IDs. Light grey segments represent metabolites without KEGG annotation. Colored rows represent different trait classes, including liver-related traits (L), abdominal fat-related traits (AF), egg yolk-related traits (EY), and body weight-related traits (BW).

To clarify the relationships between metabolite levels and fat deposition-related traits, we performed Spearman rank correlation (SRC) analyses between metabolites from the liver, duodenum, ileum, cecum, and serum and the 22 fat deposition-related phenotypes. The results showed marked tissue differences in the distribution of trait categories and the intersection patterns of significantly correlated metabolites (Figure 3A–E; *P*_adj_ < 0.05, FDR correction; Supplementary Table S4). Across all tissues, the significant correlations showed correlation coefficients (*r*) ranging from −0.61 to 0.68. Overall, the absolute *r* values were generally modest, with a mean of 0.25 and a median of 0.23. Only eight correlations had an absolute *r* value greater than 0.60, accounting for approximately 0.10% of all significant correlations (Supplementary Table S4). The liver contained the largest number of significantly correlated unique metabolites (*n* = 1520), followed by the ileum (*n* = 677) (Figure 3A,C). Fewer significantly correlated unique metabolites were detected in the duodenum, cecum, and serum, with 227, 252, and 262 metabolites, respectively; nevertheless, their distributions across L, AF, EY, and BW trait classes remained tissue dependent (Figure 3B,D,E). Compared with the duodenum, cecum, and serum, the liver and ileum contained larger numbers of metabolites significantly associated with AF, with 562 and 401 metabolites, respectively (Figure 3A,C). In contrast, the overall number of metabolites significantly associated with EY was relatively small, and these metabolites were mainly detected in the liver and serum (Figure 3A,E). Notably, the overlap between AF- and EY-associated metabolites was mainly concentrated in the liver, comprising 47 shared metabolites, eight of which were annotatable. These results indicate broad but generally modest associations between multi-tissue metabolites and fat deposition-related phenotypes, with liver and ileal metabolomic profiles showing relatively stronger phenotype-associated patterns. Accordingly, these correlation results should be interpreted primarily as phenotype-associated metabolic patterns rather than strong predictive or mechanistic relationships for individual metabolites.

**Figure 4:**
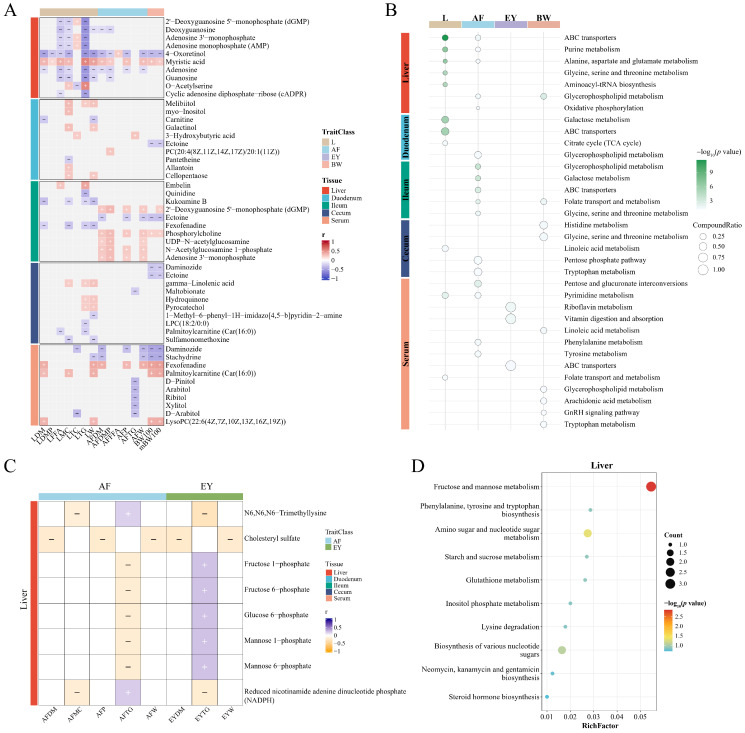
Representative metabolites and enriched pathways associated with fat deposition-related phenotypes. (**A**) Heatmap showing representative KEGG-annotated metabolites significantly correlated with fat deposition-related phenotypes across tissues. Color intensity represents Spearman correlation coefficients (r), with red indicating positive correlations and blue indicating negative correlations. “+” indicates a significant positive correlation (*P*_adj_ < 0.05), whereas “−” indicates a significant negative correlation (*P*_adj_ < 0.05). The top annotation bar indicates phenotype categories: liver-related traits (L), abdominal fat-related traits (AF), egg yolk-related traits (EY), and body weight-related traits (BW). The left annotation bar indicates tissue origin. (**B**) KEGG enrichment analysis of significantly correlated metabolites stratified by tissue and trait class after FDR correction. Dot size represents the Compound Ratio, and color indicates enrichment significance, shown as −log_10_ (FDR-adjusted *p* value). (**C**) Heatmap of metabolites significantly associated with both AF and EY traits within the same tissue. Colors represent Spearman correlation coefficients, and “+” and “−” indicate significant positive and negative correlations, respectively. (**D**) KEGG enrichment of AF–EY shared metabolites. Dot size represents the metabolite count within each enriched pathway, and dot color represents −log_10_ (FDR-adjusted *p* value).

At the metabolite level, representative KEGG-annotated metabolites showed both tissue- and trait-specific association patterns (Figure 4A), suggesting metabolite–phenotype relationships are shaped by both trait class and tissue context. KEGG enrichment analysis of significantly correlated metabolites revealed several recurrent functional themes across tissues and trait classes (Figure 4B; Supplementary Table S5). ABC transporters were repeatedly enriched in the liver, duodenum, ileum, and serum (Figure 4B), highlighting metabolite transport as a shared process associated with fat deposition-related phenotypes. Considering the potential shared metabolic basis between abdominal fat deposition and egg yolk lipid deposition, we further screened metabolites that were significantly associated with both AF and EY categories within the same tissue. Specifically, AF–EY shared metabolites were defined as metabolites significantly associated with at least one AF-related trait and at least one EY-related trait within the same tissue after FDR correction (*P*_adj_ < 0.05). AF–EY shared metabolites were mainly concentrated in the liver and included N6,N6,N6-trimethyllysine, cholesteryl sulfate, multiple sugar phosphate metabolites, and NADPH. Most of these metabolites showed opposite association patterns between abdominal fat- and egg yolk-related phenotypes (Figure 4C). KEGG enrichment analysis of AF–EY shared metabolites showed that they were mainly enriched in fructose and mannose metabolism and amino sugar and nucleotide sugar metabolism, with fructose and mannose metabolism showing the most prominent enrichment (Figure 4D). This suggested that carbohydrate metabolism and phosphorylated intermediates may represent potential components of the shared metabolic network between abdominal fat deposition and egg yolk lipid deposition.

### 3.4. Bidirectional GSMR Analysis Prioritizes Multi-Tissue Candidate Metabolites Associated with Fat Deposition-Related Traits

**Figure 5:**
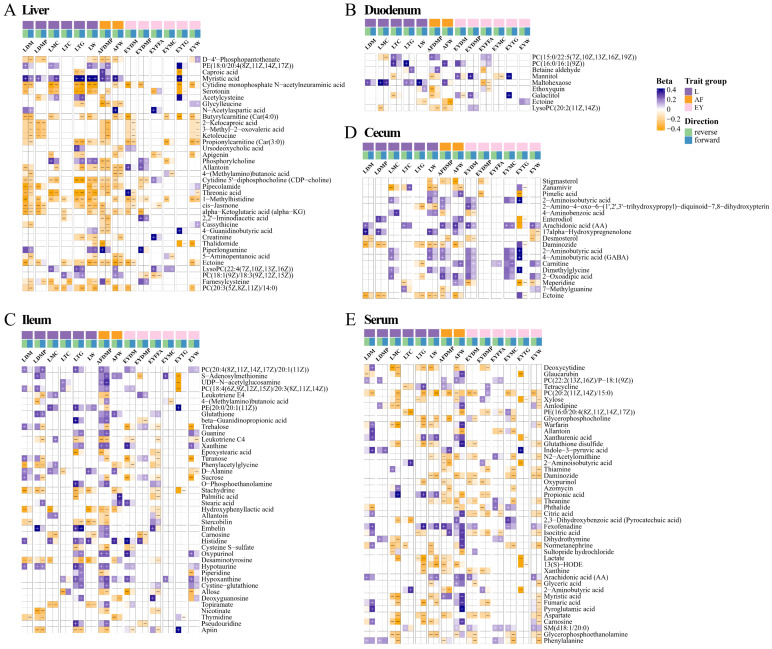
Bidirectional GSMR identifies tissue-specific candidate metabolites shared across liver, abdominal fat, and egg yolk-related traits. (**A**–**E**) Heatmaps showing GSMR effect estimates for candidate metabolites associated with liver-related traits (L), abdominal fat-related traits (AF), and egg yolk-related traits (EY) in the liver (**A**), duodenum (**B**), ileum (**C**), cecum (**D**), and serum (**E**). Only metabolites shared across the L, AF, and EY trait groups within the same tissue are shown. Columns represent phenotypes in the reverse and forward GSMR directions, and rows represent metabolites. The top annotation bars indicate trait group and GSMR analysis direction. Reverse denotes the genetically inferred directional association from metabolite abundance to the phenotype, whereas forward denotes the genetically inferred directional association from the phenotype to metabolite abundance. Tile color indicates the GSMR beta estimate. Positive GSMR effect estimates (beta > 0) indicate positive genetically inferred associations, whereas negative estimates (beta < 0) indicate negative genetically inferred associations. “+” and “−” denote significant positive and negative effects, respectively (*p* < 0.05).

To investigate genetically inferred directional associations between fat deposition-related traits and metabolites, we performed phenotype–metabolite bidirectional GSMR analyses across the liver, duodenum, ileum, cecum, and serum. A large number of phenotype–metabolite associations reaching nominal significance (*p* < 0.05) were detected across all five tissues, and the complete GSMR effect estimates, standard errors, and *p* values are provided in Supplementary Table S6. Although the associated metabolites were widely distributed across tissues, KEGG annotation coverage remained limited, with 191 of 840 liver metabolites (22.7%), 151 of 808 duodenal metabolites (18.7%), 211 of 1128 ileal metabolites (18.7%), 199 of 1025 cecal metabolites (19.4%), and 259 of 1228 serum metabolites (21.1%) annotated with KEGG IDs (Supplementary Figure S1A). Bidirectional GSMR revealed marked tissue and trait-class specificity among candidate metabolites (Supplementary Figure S1B). At the trait-class level, the largest overlap of GSMR-prioritized metabolite–tissue pairs was observed between EY- and L-related traits. Cross-tissue comparison showed that only a small subset of associated metabolites was shared across all five tissues (Supplementary Figure S1B). When focusing on metabolites shared by abdominal fat-, egg yolk-, and liver-related traits, only liver contained metabolites showing bidirectionally significant associations across all three trait classes, namely Ectoine, Cytidine 5′-diphosphocholine (CDP-choline), and Phosphorylcholine (Figure 5A–E). Ectoine and CDP-choline showed predominantly negative bidirectional associations across multiple abdominal fat- and liver-related traits, whereas phosphorylcholine showed negative bidirectional associations with AFDMP but positive bidirectional associations with EYDMP and LW (Figure 5A). Outside the liver, no metabolite showed bidirectionally significant associations spanning all three trait categories, although tissue-specific signals were still evident (Figure 5D).

To further focus on the potential metabolic partitioning relationship between abdominal fat deposition and egg yolk lipid deposition, we identified 20 shared candidate metabolites in the bidirectional GSMR analysis. These shared candidate metabolites were defined as metabolites showing nominally significant GSMR-prioritized associations (*p* < 0.05) with at least one AF-related trait and at least one EY-related trait within the same tissue, together with opposite estimated association directions between the two trait classes. Among them, seven candidate metabolites were detected in the liver and seven in serum, whereas three, two, and one candidates were detected in the duodenum, ileum, and cecum, respectively (Figure 5A–E; Supplementary Table S7). Representative metabolites included allantoin, caproic acid, CDP-choline, phosphorylcholine, and PC(18:1/18:3) in the liver (Figure 5A); galactitol, maltohexaose, and mannitol in the duodenum (Figure 5B); S-adenosylmethionine and UDP-N-acetylglucosamine in the ileum (Figure 5C); and 2-aminobutyric acid, 2-aminoisobutyric acid, N2-acetylornithine, phenylalanine, theanine, and xylose in serum (Figure 5E). For example, in the reverse GSMR direction, liver phosphorylcholine showed a negative estimated association with AFDMP (β = −0.292, SE = 0.094, *p* = 0.0019) but a positive estimated association with EYDMP (β = 0.242, SE = 0.113, *p* = 0.032). Similarly, liver allantoin showed negative estimated associations with AF traits, including AFW (β = −0.245, SE = 0.104, *p* = 0.018), but positive estimated associations with EY traits, including EYDM (β = 0.317, SE = 0.145, *p* = 0.028) and EYDMP (β = 0.270, SE = 0.128, *p* = 0.035). Liver CDP-choline also showed opposite estimated association directions between AFDMP (β = −0.170, SE = 0.073, *p* = 0.020) and EYFFA (β = 0.205, SE = 0.094, *p* = 0.030). Among these 20 candidate metabolites, liver-derived candidates showed relatively stronger genetic signals. The heritability of cytidine 5′-diphosphocholine (CDP-choline), 2,2′-iminodiacetic acid, and 4-guanidinobutyric acid reached statistical significance (*p* < 0.05), with corresponding *h*^2^ estimates of approximately 0.40, 0.41, and 0.34, respectively (Supplementary Table S3). Maltohexaose in the duodenum showed suggestive heritability (*h*^2^ = 0.24, *p* = 0.068; Supplementary Table S3). Most sugar phosphate metabolites, NADPH, and N6,N6,N6-trimethyllysine had *h*^2^ estimates close to zero, whereas only cholesteryl sulfate showed a relatively higher but non-significant heritability estimate (*h*^2^ = 0.20, *p* = 0.111; Supplementary Table S3). These results suggest that some candidate metabolites with opposite effects on abdominal fat and egg yolk lipid deposition may be under genetic regulation, whereas most shared correlated metabolites are more likely to reflect trait-associated metabolic state changes. Together, these candidate metabolites represent biologically relevant processes, including phosphatidylcholine biosynthesis, purine and uric acid metabolism, one-carbon metabolism, amino sugar metabolism, and amino acid-related metabolism, suggesting that the statistical associations may reflect metabolic processes involved in lipid synthesis, transport, and tissue-specific lipid allocation.

### 3.5. Association Networks and Core Pathways of Shared Metabolites Related to Abdominal Fat and Egg Yolk Lipid Deposition

**Figure 6:**
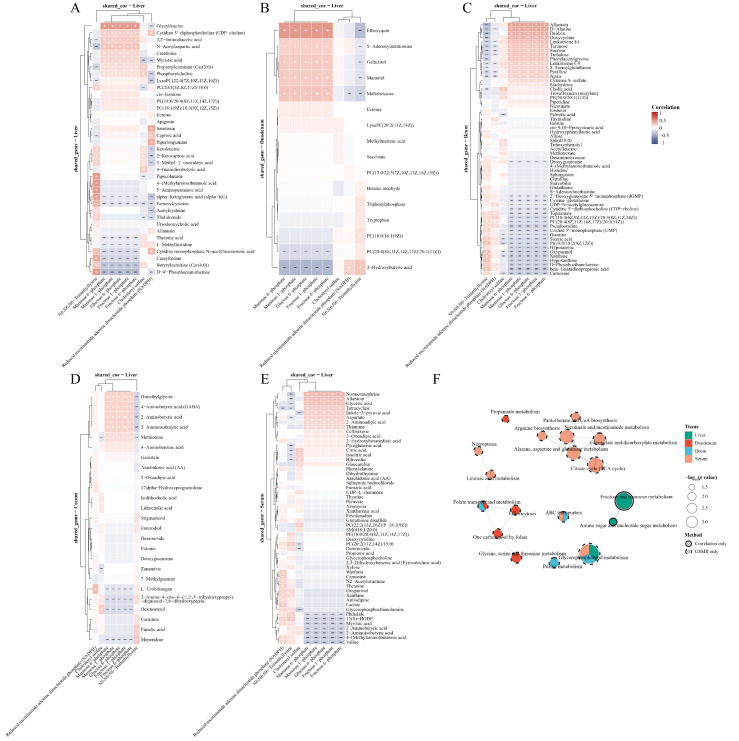
Metabolic association network and pathway convergence of AF–EY shared metabolites. (**A**–**E**) Heatmaps showing correlations between liver-derived AF–EY shared metabolites identified by Spearman rank correlation (SRC) analysis and AF–EY shared candidate metabolites identified by bidirectional GSMR analysis in the liver (**A**), duodenum (**B**), ileum (**C**), cecum (**D**), and serum (**E**). Columns represent AF–EY shared metabolites identified by SRC in the liver, and rows represent AF–EY shared candidate metabolites identified by bidirectional GSMR analysis in each tissue. Tile color indicates the correlation coefficient. “+” and “−” denote significant positive and negative correlations, respectively (*p* < 0.05). (**F**) KEGG pathway enrichment network of AF–EY shared metabolites identified from SRC and bidirectional GSMR analyses. Nodes represent enriched KEGG pathways, and edges indicate metabolite-set similarity between pathways based on the Jaccard index. Node size represents enrichment significance, shown as −log10 (FDR-adjusted *p* value). Pie colors indicate the tissue sources of metabolites contributing to each pathway. Node border type indicates whether the pathway was identified from SRC analysis only, bidirectional GSMR analysis only, or both analyses.

To further investigate the relationships among AF–EY shared metabolic signals, we correlated the abundance of eight metabolites identified by SRC with that of candidate metabolites identified by bidirectional GSMR in each tissue (Figure 6A–E). The results revealed broad correlation structures between these two classes of shared metabolites, which formed clear positive and negative correlation modules in each tissue. In the liver, AF–EY shared metabolites identified by SRC, including NADPH, sugar phosphate metabolites, and cholesteryl sulfate, were significantly correlated with candidate metabolites identified by bidirectional GSMR analysis, including CDP-choline, phosphorylcholine, myristic acid, creatinine, and allantoin (Figure 6A). Numerous significant correlations were also observed in the ileum and serum, particularly clustered correlation patterns between sugar phosphate metabolites and multiple nucleotide-, amino acid-, and lipid-related metabolites (Figure 6C,E). These results suggest that AF–EY shared correlated metabolites in the liver may together form a cross-tissue metabolic association network that includes multi-tissue candidate metabolites. Some candidate metabolites were significantly positively or negatively correlated with sugar phosphate metabolites, NADPH, and cholesteryl sulfate, suggesting that AF–EY candidate metabolites identified by bidirectional GSMR and liver shared correlated metabolites may belong to related metabolic modules involved in lipid partitioning between abdominal fat and egg yolk lipid deposition. Specifically, liver-derived candidate metabolites, including CDP-choline, phosphorylcholine, and 2,2′-iminodiacetic acid, as well as duodenal galactitol, maltohexaose, and mannitol, were significantly positively correlated with sugar phosphate metabolites. In contrast, UDP-N-acetylglucosamine in the ileum and 2-aminobutyric acid and 2-aminoisobutyric acid in serum were significantly negatively correlated with sugar phosphate metabolites.

At the pathway level, we further compared KEGG enrichment patterns between the two classes of AF–EY shared metabolites and constructed an enrichment network based on metabolite-set similarity among pathways (Figure 6F; Supplementary Table S8). The results showed that AF–EY shared metabolites were mainly enriched in fructose and mannose metabolism, glycerophospholipid metabolism, ABC transporters, folate transport and metabolism, glycine, serine and threonine metabolism, alanine, aspartate and glutamate metabolism, and the citrate cycle (TCA cycle) (Figure 6F). Fructose and mannose metabolism showed a similar connection with amino sugar and nucleotide sugar metabolism, suggesting that carbohydrate metabolism and sugar phosphate intermediates are important components of AF–EY shared metabolic signals. Glycerophospholipid metabolism was a pathway shared across the liver, ileum, and serum (Figure 6F), highlighting its potential role in the multi-tissue AF–EY shared metabolic network. In addition, folate/one-carbon metabolism, amino acid metabolism, linoleic acid metabolism, and energy metabolism-related pathways formed several local functional modules (Figure 6F). Together, these findings indicate that pathway enrichment of AF–EY shared metabolites primarily converges on core processes related to carbohydrate metabolism, glycerophospholipid metabolism, one-carbon/folate metabolism, amino acid metabolism, and energy metabolism.

## 4. Discussion

By integrating whole-genome resequencing data with metabolomic data from five tissues, this study characterized the proportion of phenotypic variance in fat deposition-related traits during the extended laying period explained by metabolites and the underlying genetic basis, and identified a series of metabolites and pathways showing genetically inferred directional associations with abdominal fat and egg yolk lipid deposition.

Although significant metabolite–phenotype correlations were widely detected, their magnitudes were generally weak to moderate. This may partly reflect the complex and polygenic nature of fat deposition-related traits, in which individual metabolites are unlikely to exert large independent effects but may instead contribute as part of coordinated multi-tissue metabolic networks. Therefore, these correlation results should be interpreted mainly as phenotype-associated metabolic patterns. Their biological relevance is better supported by the convergence of shared AF–EY association patterns, pathway enrichment, metabolite heritability, and GSMR-prioritized directional associations, rather than by the magnitude of any single metabolite–phenotype correlation.

During the laying period, estrogen drives hepatic lipoprotein assembly from generic VLDL toward yolk-targeted VLDLy. VLDLy particles are smaller and triglyceride-rich, and represent the major source of yolk lipids [9,10]. In parallel, because de novo fatty acid synthesis in birds occurs mainly in the liver, adipose tissue growth and fattening depend largely on the availability of circulating triglycerides transported by lipoproteins, particularly VLDL [5,11]. Therefore, hepatic lipid metabolic status is mechanistically linked to both abdominal fat deposition and egg yolk lipid content. In the present study, the liver metabolome showed the highest metabolite-explained variance (*me*^2^) and heritability (*h*^2^) for hepatic lipid, abdominal fat, and body weight-related traits, which is consistent with an important role of the liver in systemic lipid partitioning. Previous studies have shown that dietary supplementation with mulberry branch fiber [12], L-carnitine plus yeast chromium [34], rutin [35], or choline [36] can reduce hepatic lipid accumulation and abdominal fat deposition in laying hens, while also modulating egg yolk lipid composition. These findings suggest that lipid metabolic interventions may shift lipid allocation among hepatic storage, peripheral fat deposition, and yolk lipid deposition. The metabolites identified in this study with opposite effect directions between abdominal fat and egg yolk traits provide metabolite-level clues for this partitioning relationship.

Hepatic sugar phosphate metabolites and NADPH, as representative components of the AF–EY shared metabolic network, are closely linked to carbohydrate flux and reducing-power supply for de novo fatty acid synthesis [5,37,38,39]. Their opposite association patterns with AFTG and EYTG suggest a potential role in lipid partitioning between abdominal fat storage and egg yolk lipid deposition. N6,N6,N6-trimethyllysine is a precursor for carnitine, which plays a central role in the mitochondrial transport of long-chain fatty acids and subsequent β-oxidation [40,41]. L-carnitine supplementation has been reported to affect abdominal fat deposition, egg quality, and egg yolk lipid composition in laying hens [42,43,44]. The simultaneous association of N6,N6,N6-trimethyllysine with abdominal fat- and egg yolk-related traits in this study suggests that carnitine metabolism may be related to lipid allocation between abdominal fat storage and egg yolk deposition. Of particular interest, CDP-choline and phosphorylcholine are key intermediates in the phosphatidylcholine (PC) biosynthetic pathway [45]. Both metabolites exhibited genetically inferred directional associations with liver, abdominal fat, and egg yolk traits, but with opposite estimated effects on abdominal fat versus egg yolk. Phosphatidylcholine is an essential component of lipoprotein assembly and secretion [46], and is therefore important for hepatic triglyceride export and egg yolk lipid deposition. Previous evidence indicates that choline supplementation can increase egg yolk total lipid and phosphatidylcholine, elevate serum VLDL, and reduce hepatic lipid/triglyceride, supporting a role for choline/phosphatidylcholine metabolism in hepatic lipid export and yolk lipid deposition [36]. In addition, allantoin, a product of uric acid metabolism, has recently been shown to exacerbate hepatic lipid accumulation by inhibiting PPARα activity, thereby promoting the progression of metabolic dysfunction-associated steatotic liver disease (MASLD) [47]. In this study, the opposite GSMR-inferred directional associations of allantoin with abdominal fat and egg yolk traits suggest a role in differential lipid allocation. Previous studies have investigated purine metabolism and uric acid synthesis in chicken hepatocytes [48], as well as their genetic basis [49]. Accordingly, the signals involving allantoin and purine/pyrimidine metabolism in the present study suggest that nucleotide metabolism may not only be a marker of cellular metabolic activity, but also be linked to lipid synthesis, redox status, and hepatic metabolic burden.

Pathway analysis consistently implicated multiple metabolic processes—including sugar, glycerophospholipid, amino acid, and energy metabolism—that are known to support lipid synthesis, substrate transport, and redox balance [46,50,51,52]. Previous studies have linked egg yolk fat deposition to glycerophospholipid and one-carbon metabolism (e.g., choline, folate and vitamin B12), and the latter can influence yolk phospholipid composition and lipid metabolism in laying hens [22,53]. Notably, ABC transporters were repeatedly enriched across multiple tissues and trait classes; given their role in mediating the transport of diverse substrates, particularly lipophilic molecules such as phospholipids, bile acids, and sterols [54]; this pathway may participate in the inter-tissue transport and allocation of lipid-related metabolites during abdominal fat and egg yolk lipid deposition. The shared enrichment of glycerophospholipid metabolism in liver, ileum, and serum further suggests its potential involvement in the multi-tissue metabolic network linking abdominal fat and egg yolk lipid deposition. From an applied perspective, the metabolites and pathways identified here may serve as potential biomarkers or metabolic indicators for improving lipid allocation in laying hens. Candidate metabolites with genetic contribution or GSMR-prioritized directional associations, such as CDP-choline, phosphorylcholine, and allantoin, could be further validated and integrated with genomic information to support selection for reduced abdominal fat deposition while maintaining favorable egg yolk lipid deposition. Pathways related to glycerophospholipid, carbohydrate, and one-carbon/folate metabolism may also inform future nutritional or metabolic interventions. The moderate sample size should also be considered when interpreting the statistical power of this study. It provided useful power to detect broad tissue-level metabolomic patterns and moderate-to-large metabolite-explained variance or heritability signals, but may have limited power for weak individual metabolite–phenotype correlations, low-heritability metabolites, and subtle tissue-specific effects. For bidirectional GSMR, statistical power was additionally limited by the number and strength of available instrumental variants for each metabolite. In addition, laying rate, individual health status, and gut microbiota were not explicitly included in the current analysis. These factors may influence nutrient allocation and metabolic status, thereby contributing to variation in abdominal fat traits, egg yolk lipid deposition, and multi-tissue metabolomic profiles. Future studies should incorporate production records, health indicators, and microbiome data as covariates or jointly model them in integrative multi-omics analyses to further disentangle these effects. Nevertheless, the GSMR results should be interpreted as genetically inferred directional associations rather than definitive evidence of biological causality. Because this study was based on a moderate-sized, single purebred Rhode Island Red population at 100 weeks of age, further validation across breeds, genetic backgrounds, ages, and production systems, together with functional and controlled intervention experiments, is needed before practical application.

## 5. Conclusions

This study integrated whole-genome resequencing and multi-tissue metabolomic profiling to characterize the metabolic basis of fat deposition-related traits in laying hens during the extended laying period. Liver and serum metabolomic profiles showed broad explanatory power for liver lipid deposition, abdominal fat deposition, and body weight-related traits, whereas egg yolk-related traits displayed more restricted metabolic associations. By integrating correlation analysis, metabolite heritability, and bidirectional GSMR, we identified liver-derived sugar phosphate metabolites, NADPH, cholesteryl sulfate, N6,N6,N6-trimethyllysine, CDP-choline, phosphorylcholine and allantoin as candidate metabolic signatures associated with abdominal fat and egg yolk lipid deposition. At the pathway level, carbohydrate metabolism, glycerophospholipid metabolism, ABC transporters, folate/one-carbon metabolism, amino acid metabolism, and energy metabolism emerged as core processes underlying lipid allocation. These findings provide potential metabolic indicators for reducing excessive abdominal fat while maintaining favorable egg yolk lipid deposition in laying hens. Future studies should validate these candidate metabolites across diverse laying hen breeds and genetic backgrounds, and evaluate whether targeted dietary or metabolic interventions based on these pathways can improve lipid allocation under practical production conditions.

## Data Availability

The original contributions presented in this study are included in the article and Supplementary Materials. Further inquiries can be directed to the corresponding author.

## Supplementary Materials

The following supporting information can be downloaded at: https://www.mdpi.com/article/10.3390/ani16121812/s1, Figure S1: KEGG annotation and overlap distribution of metabolites identified by bidirectional GSMR analyses across tissues and trait groups; Table S1: Descriptive statistics for measured traits; Table S2: Proportion of total phenotypic variance explained by metabolite expression profiles across five tissues; Table S3: Heritability (h2) estimates of metabolites across tissues; Table S4: Spearman rank correlation analysis of associations between metabolite levels and all phenotypes across tissues; Table S5: KEGG pathway enrichment analysis based on the significant metabolites selected from SRC analysis; Table S6: Estimated effects of metabolites from five tissues on all phenotypes in the bidirectional GSMR analysis; Table S7: Metabolites showing differential GSMR-prioritized associations with abdominal fat- and egg yolk-related traits. Table S8. KEGG pathway enrichment analysis based on AF–EY shared significant metabolites identified from SRC and bidirectional GSMR analyses.
