# Supplementary figures and images for: Multi-Tissue Metabolomics Reveals Metabolic Signatures Associated with Lipid Partitioning Between Abdominal Fat and Egg Yolk in Laying Hens
Source: Animals (Basel). 2026 Jun 11;16(12):1812. doi: 10.3390/ani16121812 (PMC13295956; doi:10.3390/ani16121812)

A

Numbers of KEGG-annotated and non-annotated metabolites across tissues

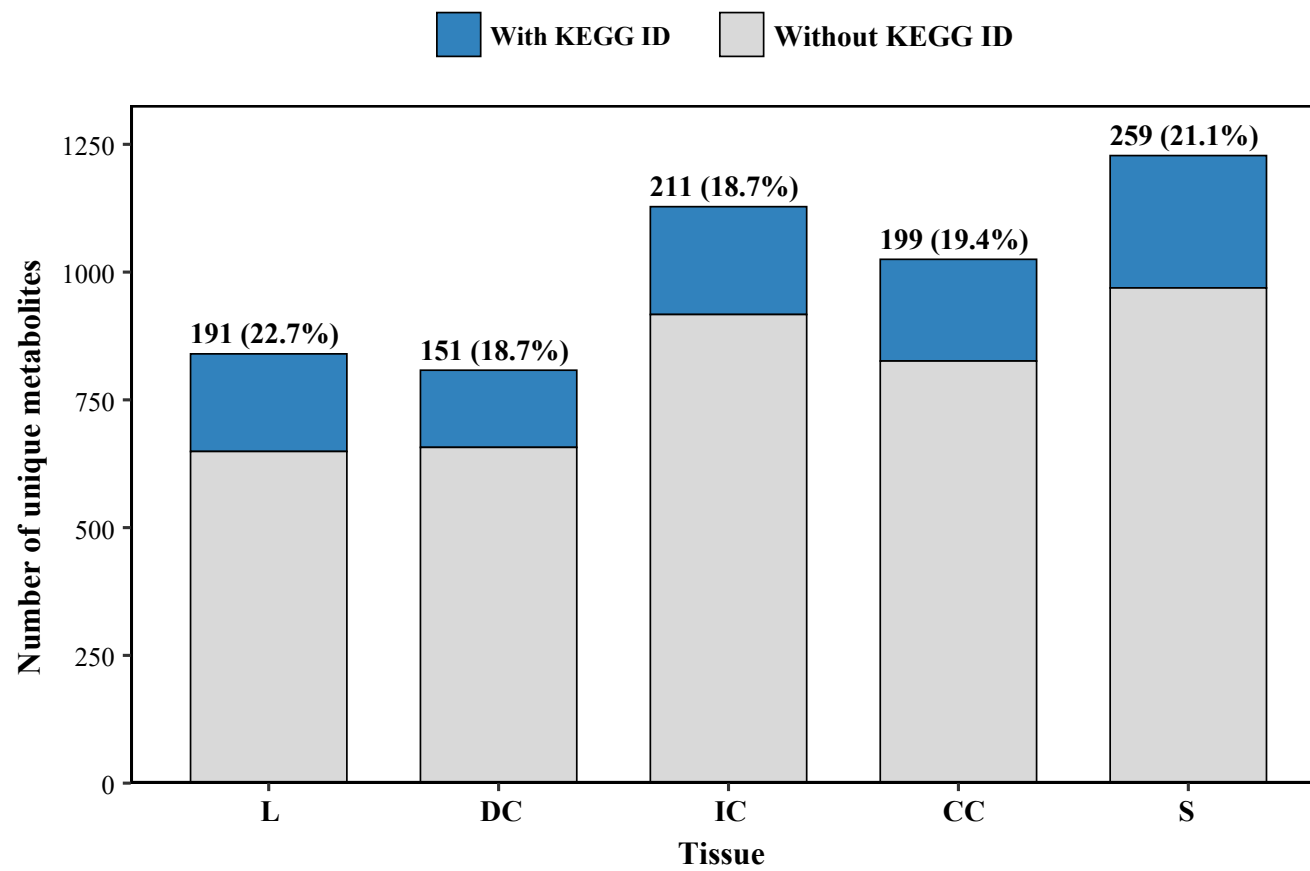

B

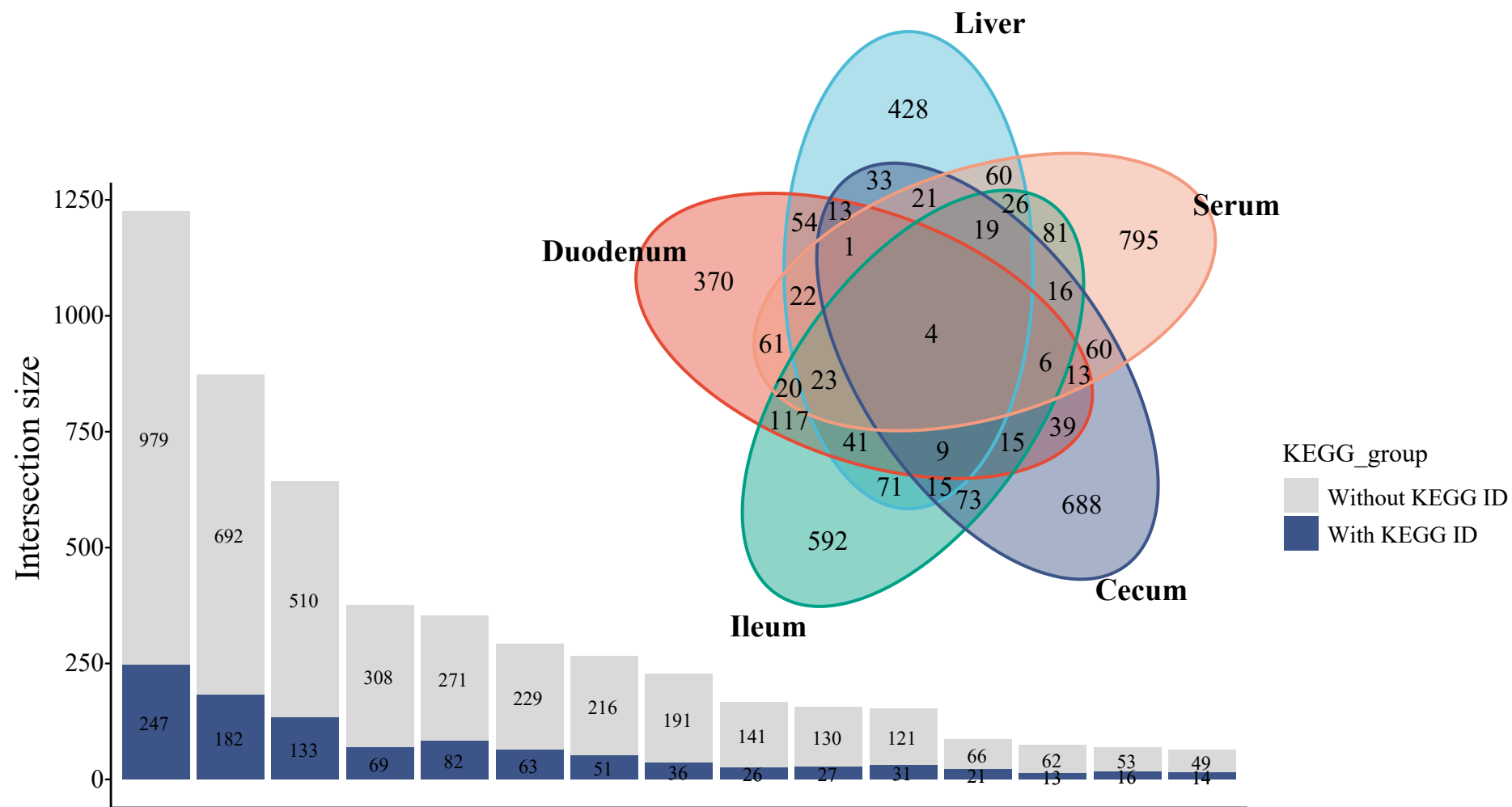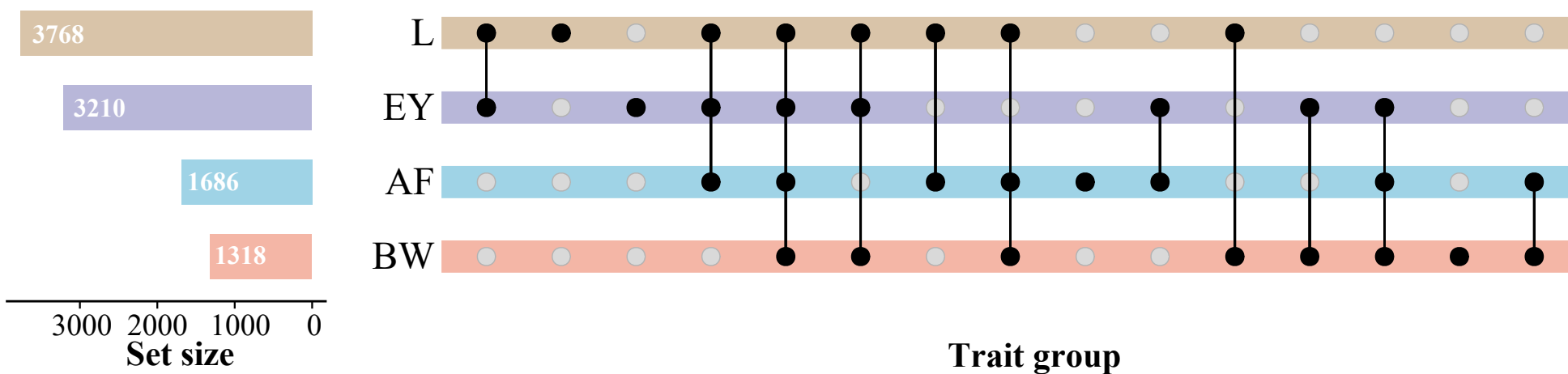

Supplement: Supplementary file 1 [file animals-16-01812-s001.zip › FigS1.pdf]
